# Supplementary material for: Analysis of influencing factors of no/low response to preoperative concurrent chemoradiotherapy in locally advanced rectal cancer
Source: PLoS One. 2020 Jun 10;15(6):e0234310. doi: 10.1371/journal.pone.0234310 (PMC7286508; doi:10.1371/journal.pone.0234310)
Supplement: Attachment — Submitted filename: Response to Reviewers.docx [file pone.0234310.s002.docx]

Dear professor:

Thank you very much for your suggestion and guidance on our manuscript. After consulting the relevant literatures, we have revised our paper according to your suggestions and questions. Our answer as follows:

**Response to Editor:**

Our answer to question one *( In the abstract, "15 factors" is better to be written as "Fifteen factors..."*. In the abstract, "15 factors" is better to be written as "Fifteen factors...". In the abstract, "15 factors" is better to be written as "Fifteen factors..."*Thank you for revising the article according the suggestions of statistical reviewer. And, the statistical reviewer would like the authors to check the relationship between the results of Tables 4 and 5, and the logistic regression models described in Results. I think Tables 4 and 5 showed the prediction of pCR based on variables listed in Tables, however, P=logistic regression model in Results showed the formula of prediction of not pCR. How do the authors think?Thank you for revising the article according the suggestions of statistical reviewer. And, the statistical reviewer would like the authors to check the relationship between the results of Tables 4 and 5, and the logistic regression models described in Results. I think Tables 4 and 5 showed the prediction of pCR based on variables listed in Tables, however, P=logistic regression model in Results showed the formula of prediction of not pCR. How do the authors think?)* is as follows:

Thank you very much for your advice. In the abstract section, we have modified this error.

Our answer to question two *(In the letter to the editor, "myco-authors" should be "my co-authors."*Thank you very much for your revision.
However, as mentioned above, our statistical advisor still has a small question. Can you check it carefully?
I also ask you to take care of significant digits again, especially in results section and tables.
Example: Materials and methods, Volumetric imaging parameters, line 4
rs=0.989
Why you show us three digits? Is there any meaning in the last digit “9”?*Thank you for revising the article according the suggestions of statistical reviewer. And, the statistical reviewer would like the authors to check the relationship between the results of Tables 4 and 5, and the logistic regression models described in Results. I think Tables 4 and 5 showed the prediction of pCR based on variables listed in Tables, however, P=logistic regression model in Results showed the formula of prediction of not pCR. How do the authors think?Thank you for revising the article according the suggestions of statistical reviewer. And, the statistical reviewer would like the authors to check the relationship between the results of Tables 4 and 5, and the logistic regression models described in Results. I think Tables 4 and 5 showed the prediction of pCR based on variables listed in Tables, however, P=logistic regression model in Results showed the formula of prediction of not pCR. How do the authors think?)* is as follows:

Thank you very much for your reminding. In the letter to the editor, we have modified this error.

Our answer to question three *(Line 68, "CD44v6," should not be Italic here. It is supposed to be a protein stained by an IHC method. Please make sure all the CD44v6 in the manuscript has been correctly expressed.* Thank you very much for your revision.
However, as mentioned above, our statistical advisor still has a small question. Can you check it carefully?
I also ask you to take care of significant digits again, especially in results section and tables.
Example: Materials and methods, Volumetric imaging parameters, line 4
rs=0.989
Why you show us three digits? Is there any meaning in the last digit “9”?*Thank you for revising the article according the suggestions of statistical reviewer. And, the statistical reviewer would like the authors to check the relationship between the results of Tables 4 and 5, and the logistic regression models described in Results. I think Tables 4 and 5 showed the prediction of pCR based on variables listed in Tables, however, P=logistic regression model in Results showed the formula of prediction of not pCR. How do the authors think?Thank you for revising the article according the suggestions of statistical reviewer. And, the statistical reviewer would like the authors to check the relationship between the results of Tables 4 and 5, and the logistic regression models described in Results. I think Tables 4 and 5 showed the prediction of pCR based on variables listed in Tables, however, P=logistic regression model in Results showed the formula of prediction of not pCR. How do the authors think?)* is as follows:

Thank you very much for your reminding. We carefully checked the entire manuscript and made comprehensive corrections to the writing errors of CD44v6. Thanks again for your advice.

Our answer to question four *(Line 69-70, "Breast cancer specimens were used to act as positive control samples, PBS (pH 7.4) was used as a negative control" They should be two different sentences.* Thank you very much for your revision.
However, as mentioned above, our statistical advisor still has a small question. Can you check it carefully?
I also ask you to take care of significant digits again, especially in results section and tables.
Example: Materials and methods, Volumetric imaging parameters, line 4
rs=0.989
Why you show us three digits? Is there any meaning in the last digit “9”?*Thank you for revising the article according the suggestions of statistical reviewer. And, the statistical reviewer would like the authors to check the relationship between the results of Tables 4 and 5, and the logistic regression models described in Results. I think Tables 4 and 5 showed the prediction of pCR based on variables listed in Tables, however, P=logistic regression model in Results showed the formula of prediction of not pCR. How do the authors think?Thank you for revising the article according the suggestions of statistical reviewer. And, the statistical reviewer would like the authors to check the relationship between the results of Tables 4 and 5, and the logistic regression models described in Results. I think Tables 4 and 5 showed the prediction of pCR based on variables listed in Tables, however, P=logistic regression model in Results showed the formula of prediction of not pCR. How do the authors think?)* is as follows:

Thank you very much for your advice. We have modified this error accordingly in our revised version.

Our answer to question five *(The authors said that they used breast cancer samples for control. Was the breast cancer sample prospectively taken or a control slide? Otherwise, please make a clear statement of how the authors used breast cancer samples within the approval range of the IRB.* Thank you very much for your revision.
However, as mentioned above, our statistical advisor still has a small question. Can you check it carefully?
I also ask you to take care of significant digits again, especially in results section and tables.
Example: Materials and methods, Volumetric imaging parameters, line 4
rs=0.989
Why you show us three digits? Is there any meaning in the last digit “9”?*Thank you for revising the article according the suggestions of statistical reviewer. And, the statistical reviewer would like the authors to check the relationship between the results of Tables 4 and 5, and the logistic regression models described in Results. I think Tables 4 and 5 showed the prediction of pCR based on variables listed in Tables, however, P=logistic regression model in Results showed the formula of prediction of not pCR. How do the authors think?Thank you for revising the article according the suggestions of statistical reviewer. And, the statistical reviewer would like the authors to check the relationship between the results of Tables 4 and 5, and the logistic regression models described in Results. I think Tables 4 and 5 showed the prediction of pCR based on variables listed in Tables, however, P=logistic regression model in Results showed the formula of prediction of not pCR. How do the authors think?)* is as follows:

Thank you very much for your advice. Due to our negligence, the reason for using breast cancer samples as a positive control is not described in the manuscript. In this revised manuscript, we have shown the corresponding basis. Thank you again for your reminding.

Our answer to question six *(All the tables lack abbreviations independently. Please add them in the bottom of each tables.*Thank you very much for your revision.
However, as mentioned above, our statistical advisor still has a small question. Can you check it carefully?
I also ask you to take care of significant digits again, especially in results section and tables.
Example: Materials and methods, Volumetric imaging parameters, line 4
rs=0.989
Why you show us three digits? Is there any meaning in the last digit “9”?*Thank you for revising the article according the suggestions of statistical reviewer. And, the statistical reviewer would like the authors to check the relationship between the results of Tables 4 and 5, and the logistic regression models described in Results. I think Tables 4 and 5 showed the prediction of pCR based on variables listed in Tables, however, P=logistic regression model in Results showed the formula of prediction of not pCR. How do the authors think?Thank you for revising the article according the suggestions of statistical reviewer. And, the statistical reviewer would like the authors to check the relationship between the results of Tables 4 and 5, and the logistic regression models described in Results. I think Tables 4 and 5 showed the prediction of pCR based on variables listed in Tables, however, P=logistic regression model in Results showed the formula of prediction of not pCR. How do the authors think?)* is as follows:

Thank you very much for your reminding. We have added abbreviations in the bottom of each table in revised version.

Our answer to question seven *(No potential limitations were stated in the manuscript. Please kindly consider providing one paragraph for the limitations of the present study.*Thank you very much for your revision.
However, as mentioned above, our statistical advisor still has a small question. Can you check it carefully?
I also ask you to take care of significant digits again, especially in results section and tables.
Example: Materials and methods, Volumetric imaging parameters, line 4
rs=0.989
Why you show us three digits? Is there any meaning in the last digit “9”?*Thank you for revising the article according the suggestions of statistical reviewer. And, the statistical reviewer would like the authors to check the relationship between the results of Tables 4 and 5, and the logistic regression models described in Results. I think Tables 4 and 5 showed the prediction of pCR based on variables listed in Tables, however, P=logistic regression model in Results showed the formula of prediction of not pCR. How do the authors think?Thank you for revising the article according the suggestions of statistical reviewer. And, the statistical reviewer would like the authors to check the relationship between the results of Tables 4 and 5, and the logistic regression models described in Results. I think Tables 4 and 5 showed the prediction of pCR based on variables listed in Tables, however, P=logistic regression model in Results showed the formula of prediction of not pCR. How do the authors think?)* is as follows:

Thank you very much for your reminding. With your suggestion, we carefully read relevant literatures and conducted in-depth thinking and discussion. We found that our study does have some limitations, so we elaborated them one by one in the discussion section of the revised version.

Our answer to question eight *(In conclusion, the authors are recommended to be more humble. The findings here have not been independently validated. Please do not conclude that some patients should be "saved" from the corresponding radiation complications. The conclusion is too aggressive and not scientific enough.*Thank you very much for your revision.
However, as mentioned above, our statistical advisor still has a small question. Can you check it carefully?
I also ask you to take care of significant digits again, especially in results section and tables.
Example: Materials and methods, Volumetric imaging parameters, line 4
rs=0.989
Why you show us three digits? Is there any meaning in the last digit “9”?*Thank you for revising the article according the suggestions of statistical reviewer. And, the statistical reviewer would like the authors to check the relationship between the results of Tables 4 and 5, and the logistic regression models described in Results. I think Tables 4 and 5 showed the prediction of pCR based on variables listed in Tables, however, P=logistic regression model in Results showed the formula of prediction of not pCR. How do the authors think?Thank you for revising the article according the suggestions of statistical reviewer. And, the statistical reviewer would like the authors to check the relationship between the results of Tables 4 and 5, and the logistic regression models described in Results. I think Tables 4 and 5 showed the prediction of pCR based on variables listed in Tables, however, P=logistic regression model in Results showed the formula of prediction of not pCR. How do the authors think?)* is as follows:

Thank you very much for your advice. Your criticism is correct and we have accepted it sincerely. In the conclusion section, we have deleted and modified those inappropriate statements.

**Response to Reviewer#1:**

Our answer to question one *(The patients qualified for the therapy had upper rectal cancer (10 cm from the anal verge)-from the data we do not know, which cancers were above peritoneal reflection (>12 cm from the anal verge)-->these do not benefit from CRT and should be treated as colon cancer. In the abstract, "15 factors" is better to be written as "Fifteen factors...". In the abstract, "15 factors" is better to be written as "Fifteen factors..."Thank you for revising the article according the suggestions of statistical reviewer. And, the statistical reviewer would like the authors to check the relationship between the results of Tables 4 and 5, and the logistic regression models described in Results. I think Tables 4 and 5 showed the prediction of pCR based on variables listed in Tables, however, P=logistic regression model in Results showed the formula of prediction of not pCR. How do the authors think?Thank you for revising the article according the suggestions of statistical reviewer. And, the statistical reviewer would like the authors to check the relationship between the results of Tables 4 and 5, and the logistic regression models described in Results. I think Tables 4 and 5 showed the prediction of pCR based on variables listed in Tables, however, P=logistic regression model in Results showed the formula of prediction of not pCR. How do the authors think?)* is as follows:

Thank you very much for your reminding. With your suggestion, we carefully read relevant literatures. The relevant contents we have read are reported as follows: The rectum is defined as the distal 16 cm of the large intestine leading to the anal canal, and a cancer between 12 and 16 cm from the anal verge is classified as a rectal cancer of the upper third[1,2]. Previous studies have shown that neoadjuvant chemoradiotherapy have no significant effect on the upper rectal cancer [3, 4]. At present, most scholars believe that preoperative CCRT is necessary for tumor under the anterior peritoneal reflection. Memon 's study showed that the mean distance from the anal verge to the anterior peritoneal reflection was 11.9 cm (men) and 10 cm (women) [5]. Studies by Peeters found that radiotherapy has a significant effect on reducing the risk of local recurrence in patients with lesions between 5 and 10 cm from the anal verge [4]. In China, most scholars defined between 10 and 15 cm from the anal verge as the upper rectum, 5-10 cm from the anal verge as the middle rectum, and 5 cm above the anal verge as the lower rectum [6]. Therefore, in this study, we took the distance of less than 10cm between the inferior margin of the tumor and the anal verge less than 10cm as one of the inclusion criteria.

**References**

1. Soreide O, Norstein J, Fielding LP, et al. International standardization and documentation of the treatment of rectal cancer. In:Soreide O, Norstein J, eds. *Rectal Cancer Surgery*. Optimization-*Standardization-Documentation*. Berlin, Germany: Springer;1997:405– 445.

2. Wittekind C, Greene FL, Henson C, eds. *UICC. TNM Classification of Malignant Tumors*, 6th ed. New York, NY: John Wiley & Sons; 2002.

3. Kapiteijn E, Marijnen C, Nagtegaal ID, et al. Preoperative radiotherapy combined with total mesorectal excision for resectable rectal cancer. *N Engl J Med*. 2001;345:638–646.

4. Peeters KCMJ, Marijnen CAM, Nagtegaal ID, et al. The TME trial after a median follow-up of 6 years: increased local control but no survival benefit in irradiated patients with resectable rectal carcinoma. *Ann Surg*. 2007;246:693–701.

5. Memon S, Keating JP, Cooke HS, et al. A study into external rectal anatomy: improving patient selection for radiotherapy for rectal cancer. Dis Colon Rectum. 2009 Jan; 52(1):87-90.

6. Wen xin, LIU Lei , XIA Jin dong, et al. MRI in determining the location of rectal cancer with respect to the peritoneal reflection. J Surg Concepts Pract (china) 2013:18(5):446-449.

Our answer to question two *(in discussion there are mentioned disadvantages of CRT-->the problems with peritoneal wound healing (none of patients had peritoneal wound-->all had TME, no abdominoperineal resections), anastomotic leakage (no data about it, we do not know if the patients had protective artificial anus). In the abstract, "15 factors" is better to be written as "Fifteen factors...". In the abstract, "15 factors" is better to be written as "Fifteen factors..."Thank you for revising the article according the suggestions of statistical reviewer. And, the statistical reviewer would like the authors to check the relationship between the results of Tables 4 and 5, and the logistic regression models described in Results. I think Tables 4 and 5 showed the prediction of pCR based on variables listed in Tables, however, P=logistic regression model in Results showed the formula of prediction of not pCR. How do the authors think?Thank you for revising the article according the suggestions of statistical reviewer. And, the statistical reviewer would like the authors to check the relationship between the results of Tables 4 and 5, and the logistic regression models described in Results. I think Tables 4 and 5 showed the prediction of pCR based on variables listed in Tables, however, P=logistic regression model in Results showed the formula of prediction of not pCR. How do the authors think?)* is as follows:

Thank you for your advice. We have supplemented this part in the "Operation and Pathologic regression status after CCRT" section of the revised version, as described next: “Out of all enrolled patients, 17 underwent Miles surgery, 62 underwent Dixon surgery, none of whom underwent Hartmann surgery. Of the 62 patients who underwent Dixon surgery, 12 underwent a prophylactic terminal ileostomy”. Thank you again for your reminding.

Our answer to question three *(the factors which are the most important during surgery and in the local treatment of the rectal cancer are clear margins (circumferential-->positive in the group with low/no response to preoperative CCRT-->5 patients, negative in the group with obvious response to preoperative CCRT-->0 patients, the authors do not mention proximal and distal margins which are also important). We do not have data about MRI images after preoperative CCRT before surgery-->if the clear margins in advanced cT3-T4 are at risk (possible R1 or R2 resection), why to perform TME-->these patients should undergo multivisceral R0 resection if it is technically possible; we do not have information in which cT the margins were positive. In the abstract, "15 factors" is better to be written as "Fifteen factors...". In the abstract, "15 factors" is better to be written as "Fifteen factors..."Thank you for revising the article according the suggestions of statistical reviewer. And, the statistical reviewer would like the authors to check the relationship between the results of Tables 4 and 5, and the logistic regression models described in Results. I think Tables 4 and 5 showed the prediction of pCR based on variables listed in Tables, however, P=logistic regression model in Results showed the formula of prediction of not pCR. How do the authors think?Thank you for revising the article according the suggestions of statistical reviewer. And, the statistical reviewer would like the authors to check the relationship between the results of Tables 4 and 5, and the logistic regression models described in Results. I think Tables 4 and 5 showed the prediction of pCR based on variables listed in Tables, however, P=logistic regression model in Results showed the formula of prediction of not pCR. How do the authors think?)* is as follows:

Thank you for your reminding. Due to negligence, we did not show the situation of the proximal and distal margins in the manuscript, which caused you a lot of trouble in review, and we apologize deeply. We have supplemented this result in the "Operation and Pathologic regression status after CCRT" section of the revised version, as described next: “The proximal and distal margins of all specimens were negative.”

In addition, the MRI images (after preoperative CCRT before surgery) showed that the status of CRM of all enrolled patients was negative, while the postoperative pathology showed that the CRM of 5 patients were positive, indicating that there was an error in the preoperative MRI evaluation of CRM. Therefore, we did not show them in the original manuscript. After receiving your suggestion, we realized the importance of presenting the CRM status of MRIs, so we supplemented these information in "Table 1: baseline demographics of all patients" in the revised version. Thank you again for your reminding.

Our answer to question four *(not all cT2N+/T3 tumors need preoperative CCRT if the quality of TME is high-->we do not have any information about the quality of surgery. In the abstract, "15 factors" is better to be written as "Fifteen factors...". In the abstract, "15 factors" is better to be written as "Fifteen factors..."Thank you for revising the article according the suggestions of statistical reviewer. And, the statistical reviewer would like the authors to check the relationship between the results of Tables 4 and 5, and the logistic regression models described in Results. I think Tables 4 and 5 showed the prediction of pCR based on variables listed in Tables, however, P=logistic regression model in Results showed the formula of prediction of not pCR. How do the authors think?Thank you for revising the article according the suggestions of statistical reviewer. And, the statistical reviewer would like the authors to check the relationship between the results of Tables 4 and 5, and the logistic regression models described in Results. I think Tables 4 and 5 showed the prediction of pCR based on variables listed in Tables, however, P=logistic regression model in Results showed the formula of prediction of not pCR. How do the authors think?)* is as follows:

Thank you for your advice. At present, preoperative CCRT combined with TME as the standard treatment for LARC patients has been written into many guidelines. In clinical practice, as you said, we did experience many patients with cT2N+/T3 who did not undergo preoperative CCRT but preferred TME surgery and achieved good results. However, 79 patients enrolled in this study were given the treatment plan at the experimental design stage, so they all adopted preoperative CCRT. With your suggestion, we supplemented the information about the quality of surgery in the" Preoperative CCRT and surgery" section of the revised manuscript as follows: “Total mesorectal excision (TME) surgery was performed by two surgeons with more than 15 years of experience at 8 to 10 weeks after CCRT, each of whom completed at least 100 rectal cancer surgeries per- year”. Thank you again for your reminding.

Our answer to question five *(even the low response to the preoperative CCRT may mean R0 surgery-->the shrinkage of the tumor should result in a clear CRM (>1mm) so we should not discourage these patients from the preoperative CCRT; the pathologic regression in 24 patients meant no/low response, but only in 5 patients the CRM was positive. In the abstract, "15 factors" is better to be written as "Fifteen factors...". In the abstract, "15 factors" is better to be written as "Fifteen factors..."Thank you for revising the article according the suggestions of statistical reviewer. And, the statistical reviewer would like the authors to check the relationship between the results of Tables 4 and 5, and the logistic regression models described in Results. I think Tables 4 and 5 showed the prediction of pCR based on variables listed in Tables, however, P=logistic regression model in Results showed the formula of prediction of not pCR. How do the authors think?Thank you for revising the article according the suggestions of statistical reviewer. And, the statistical reviewer would like the authors to check the relationship between the results of Tables 4 and 5, and the logistic regression models described in Results. I think Tables 4 and 5 showed the prediction of pCR based on variables listed in Tables, however, P=logistic regression model in Results showed the formula of prediction of not pCR. How do the authors think?)* is as follows:

What you said is very reasonable. It is true that many patients did not reach the stage of pathological decline after receiving the neoadjuvant therapy, but the tumor volume was reduced, thus R0 resection was performed. What cannot be ignored, however, is that there are still some patients not only did not achieve pathological decline and tumor volume shrinkage after CCRT, but also developed further aggravation. We all know that perioperative adjuvant treatment includes preoperative treatment and postoperative treatment. If we can identify these patients who are not sensitive to preoperative CCRT before treatment (the purpose of this study), give them active surgical treatment and then carry out postoperative radiotherapy and systemic treatment, these patients may receive greater benefits.

Our answer to question six *(emotional, general expressions like "in the real world" (line 34) "a catastrophe for the patients" (line 174) should be omitted. In the abstract, "15 factors" is better to be written as "Fifteen factors...". In the abstract, "15 factors" is better to be written as "Fifteen factors..."Thank you for revising the article according the suggestions of statistical reviewer. And, the statistical reviewer would like the authors to check the relationship between the results of Tables 4 and 5, and the logistic regression models described in Results. I think Tables 4 and 5 showed the prediction of pCR based on variables listed in Tables, however, P=logistic regression model in Results showed the formula of prediction of not pCR. How do the authors think?Thank you for revising the article according the suggestions of statistical reviewer. And, the statistical reviewer would like the authors to check the relationship between the results of Tables 4 and 5, and the logistic regression models described in Results. I think Tables 4 and 5 showed the prediction of pCR based on variables listed in Tables, however, P=logistic regression model in Results showed the formula of prediction of not pCR. How do the authors think?)* is as follows:

Thank you for your advice. We have deleted these contents in the revised manuscript.

Our answer to question seven *(endorectal ultrasound is not used for diagnosis of distant metastases (line 53). In the abstract, "15 factors" is better to be written as "Fifteen factors...". In the abstract, "15 factors" is better to be written as "Fifteen factors..."Thank you for revising the article according the suggestions of statistical reviewer. And, the statistical reviewer would like the authors to check the relationship between the results of Tables 4 and 5, and the logistic regression models described in Results. I think Tables 4 and 5 showed the prediction of pCR based on variables listed in Tables, however, P=logistic regression model in Results showed the formula of prediction of not pCR. How do the authors think?Thank you for revising the article according the suggestions of statistical reviewer. And, the statistical reviewer would like the authors to check the relationship between the results of Tables 4 and 5, and the logistic regression models described in Results. I think Tables 4 and 5 showed the prediction of pCR based on variables listed in Tables, however, P=logistic regression model in Results showed the formula of prediction of not pCR. How do the authors think?)* is as follows:

Thank you for your advice. We have deleted this content in the revised manuscript. Thank you again for your reminding.

Our answer to question eight *(grammatical and structural mistakes (line 40, 47, 48, 52, 164, 165, 179, 188, 203). In the abstract, "15 factors" is better to be written as "Fifteen factors...". In the abstract, "15 factors" is better to be written as "Fifteen factors..."Thank you for revising the article according the suggestions of statistical reviewer. And, the statistical reviewer would like the authors to check the relationship between the results of Tables 4 and 5, and the logistic regression models described in Results. I think Tables 4 and 5 showed the prediction of pCR based on variables listed in Tables, however, P=logistic regression model in Results showed the formula of prediction of not pCR. How do the authors think?Thank you for revising the article according the suggestions of statistical reviewer. And, the statistical reviewer would like the authors to check the relationship between the results of Tables 4 and 5, and the logistic regression models described in Results. I think Tables 4 and 5 showed the prediction of pCR based on variables listed in Tables, however, P=logistic regression model in Results showed the formula of prediction of not pCR. How do the authors think?)* is as follows:

Thank you for your advice. With your suggestion, we invited native English speakers to proofread the language of this manuscript.

**Response to Reviewer#2:**

Our answer to question one *(Table 1 should be replaced in results. In the abstract, "15 factors" is better to be written as "Fifteen factors...". In the abstract, "15 factors" is better to be written as "Fifteen factors..."Thank you for revising the article according the suggestions of statistical reviewer. And, the statistical reviewer would like the authors to check the relationship between the results of Tables 4 and 5, and the logistic regression models described in Results. I think Tables 4 and 5 showed the prediction of pCR based on variables listed in Tables, however, P=logistic regression model in Results showed the formula of prediction of not pCR. How do the authors think?Thank you for revising the article according the suggestions of statistical reviewer. And, the statistical reviewer would like the authors to check the relationship between the results of Tables 4 and 5, and the logistic regression models described in Results. I think Tables 4 and 5 showed the prediction of pCR based on variables listed in Tables, however, P=logistic regression model in Results showed the formula of prediction of not pCR. How do the authors think?)* is as follows:

According to your opinion, we rearranged the pathological results related to tumor regression in Table 1 into the results section of this manuscript.

Our answer to question two *(English by place could be improved by a native speaker. In the abstract, "15 factors" is better to be written as "Fifteen factors...". In the abstract, "15 factors" is better to be written as "Fifteen factors..."Thank you for revising the article according the suggestions of statistical reviewer. And, the statistical reviewer would like the authors to check the relationship between the results of Tables 4 and 5, and the logistic regression models described in Results. I think Tables 4 and 5 showed the prediction of pCR based on variables listed in Tables, however, P=logistic regression model in Results showed the formula of prediction of not pCR. How do the authors think?Thank you for revising the article according the suggestions of statistical reviewer. And, the statistical reviewer would like the authors to check the relationship between the results of Tables 4 and 5, and the logistic regression models described in Results. I think Tables 4 and 5 showed the prediction of pCR based on variables listed in Tables, however, P=logistic regression model in Results showed the formula of prediction of not pCR. How do the authors think?)* is as follows:

Thank you for your advice. With your suggestion, we invited native English speakers to proofread the language of this manuscript.

Our answer to question three *(The authors should better explain in Introduction what is CD44v6 and why it could be relevant in rectal cancer irradiation. In the abstract, "15 factors" is better to be written as "Fifteen factors...". In the abstract, "15 factors" is better to be written as "Fifteen factors..."Thank you for revising the article according the suggestions of statistical reviewer. And, the statistical reviewer would like the authors to check the relationship between the results of Tables 4 and 5, and the logistic regression models described in Results. I think Tables 4 and 5 showed the prediction of pCR based on variables listed in Tables, however, P=logistic regression model in Results showed the formula of prediction of not pCR. How do the authors think?Thank you for revising the article according the suggestions of statistical reviewer. And, the statistical reviewer would like the authors to check the relationship between the results of Tables 4 and 5, and the logistic regression models described in Results. I think Tables 4 and 5 showed the prediction of pCR based on variables listed in Tables, however, P=logistic regression model in Results showed the formula of prediction of not pCR. How do the authors think?)* is as follows:

Your opinion is reasonable. We have explained CD44v6 in the introduction section of the revised version. Thank you for your advice.

Our answer to question four *(It remains unclear whether poor response was defined according to pathological examination or with preoperative MRI. In the abstract, "15 factors" is better to be written as "Fifteen factors...". In the abstract, "15 factors" is better to be written as "Fifteen factors..."Thank you for revising the article according the suggestions of statistical reviewer. And, the statistical reviewer would like the authors to check the relationship between the results of Tables 4 and 5, and the logistic regression models described in Results. I think Tables 4 and 5 showed the prediction of pCR based on variables listed in Tables, however, P=logistic regression model in Results showed the formula of prediction of not pCR. How do the authors think?Thank you for revising the article according the suggestions of statistical reviewer. And, the statistical reviewer would like the authors to check the relationship between the results of Tables 4 and 5, and the logistic regression models described in Results. I think Tables 4 and 5 showed the prediction of pCR based on variables listed in Tables, however, P=logistic regression model in Results showed the formula of prediction of not pCR. How do the authors think?)* is as follows:

Thank you for your reminding. We know that as you said, there are no unified standards for evaluating the efficacy of preoperative CCRT in patients with rectal cancer. Common methods include preoperative imaging evaluation (especially MRI) and postoperative pathology evaluation. However, these methods focus more on patients who are sensitive to neoadjuvant therapy because some of these patients may choose the "watch and wait" strategy. But even among those patients who were evaluated as cCR, some relapsed during the "watch and wait" strategy. In clinical practice, we often meet some patients who underwent MRI examination 8-10 weeks after receiving the preoperative CCRT and found that the imaging T stage did not change significantly from the original MRI results, but the postoperative pathology displayed tumor regression. Therefore, the tumor regression status indicated by MRI after CCRT and before surgery can not be used as the gold standard for evaluating the response of lesions to CCRT, but can only be used as an important reference.

Our answer to question five *(The authors report important results which could help decision making in the future. However, I suggest that an external cohort could validate these results. In the abstract, "15 factors" is better to be written as "Fifteen factors...". In the abstract, "15 factors" is better to be written as "Fifteen factors..."Thank you for revising the article according the suggestions of statistical reviewer. And, the statistical reviewer would like the authors to check the relationship between the results of Tables 4 and 5, and the logistic regression models described in Results. I think Tables 4 and 5 showed the prediction of pCR based on variables listed in Tables, however, P=logistic regression model in Results showed the formula of prediction of not pCR. How do the authors think?Thank you for revising the article according the suggestions of statistical reviewer. And, the statistical reviewer would like the authors to check the relationship between the results of Tables 4 and 5, and the logistic regression models described in Results. I think Tables 4 and 5 showed the prediction of pCR based on variables listed in Tables, however, P=logistic regression model in Results showed the formula of prediction of not pCR. How do the authors think?)* is as follows:

Thank you for your reminding. Your suggestion is very important for our follow-up work. In the follow-up work, we will not only verify it in patients with long-course preoperative CCRT, but also in patients with short-course preoperative radiotherapy. These contents are described in the Limitations section of the revised version.

Our answer to question six *(Again, to improve the manuscript, a prognostic nomogram including all relevant parameters should be proposed and would greatly improve the quality of the manuscript.. In the abstract, "15 factors" is better to be written as "Fifteen factors...". In the abstract, "15 factors" is better to be written as "Fifteen factors..."Thank you for revising the article according the suggestions of statistical reviewer. And, the statistical reviewer would like the authors to check the relationship between the results of Tables 4 and 5, and the logistic regression models described in Results. I think Tables 4 and 5 showed the prediction of pCR based on variables listed in Tables, however, P=logistic regression model in Results showed the formula of prediction of not pCR. How do the authors think?Thank you for revising the article according the suggestions of statistical reviewer. And, the statistical reviewer would like the authors to check the relationship between the results of Tables 4 and 5, and the logistic regression models described in Results. I think Tables 4 and 5 showed the prediction of pCR based on variables listed in Tables, however, P=logistic regression model in Results showed the formula of prediction of not pCR. How do the authors think?)* is as follows:

Thank you very much for your reminding. We have drawn the nomogram chart in the revised draft and elaborated it in the results and discussion section. Thanks again for your advice.

Our answer to question seven *(Possible bias should be discussed. In the abstract, "15 factors" is better to be written as "Fifteen factors...". In the abstract, "15 factors" is better to be written as "Fifteen factors..."Thank you for revising the article according the suggestions of statistical reviewer. And, the statistical reviewer would like the authors to check the relationship between the results of Tables 4 and 5, and the logistic regression models described in Results. I think Tables 4 and 5 showed the prediction of pCR based on variables listed in Tables, however, P=logistic regression model in Results showed the formula of prediction of not pCR. How do the authors think?Thank you for revising the article according the suggestions of statistical reviewer. And, the statistical reviewer would like the authors to check the relationship between the results of Tables 4 and 5, and the logistic regression models described in Results. I think Tables 4 and 5 showed the prediction of pCR based on variables listed in Tables, however, P=logistic regression model in Results showed the formula of prediction of not pCR. How do the authors think?)* is as follows:

According to your opinion, I discussed the *bias* and limitations of our study in the Discussion section of the revised version.

Thank you and best regards.

Yours sincerely,

The author of PONE-D-19-31144

February 20, 2020

From the Editor:
Although the Vice Editor-In-Chief requested the corrections of "a significant figure" in the previous decision letter, authors did not revise them in the revised manuscript.
Abstract Line 5 from bottom: …the negative predictive values of TC were 74.7347%, 94.6097%, respectively.Please keep attention to the number of significant figures.
There are only 79 patients in your study. Therefore, they should be “75 and 95 %, respectively.
In total, 62 (78.48%) patients underwent low abdominal resection, and 17 (21.52%) underwent…78.46% should be 78% 21.52% should be 22% There are many this kind of errors in the following text.
Please correct all of them carefully again.From the Editor:
Although the Vice Editor-In-Chief requested the corrections of "a significant figure" in the previous decision letter, authors did not revise them in the revised manuscript.
Abstract Line 5 from bottom: …the negative predictive values of TC were 74.7347%, 94.6097%, respectively.Please keep attention to the number of significant figures.
There are only 79 patients in your study. Therefore, they should be “75 and 95 %, respectively.
In total, 62 (78.48%) patients underwent low abdominal resection, and 17 (21.52%) underwent…78.46% should be 78% 21.52% should be 22% There are many this kind of errors in the following text.
Please correct all of them carefully again.
