# Supplementary material for: Analysis of influencing factors of no/low response to preoperative concurrent chemoradiotherapy in locally advanced rectal cancer
Source: PLoS One. 2020 Jun 10;15(6):e0234310. doi: 10.1371/journal.pone.0234310 (PMC7286508; doi:10.1371/journal.pone.0234310)
Supplement: Attachment — Submitted filename: Response to Reviewers.docx [file pone.0234310.s003.docx]

Dear professor:

Thank you very much for your suggestion and guidance on our manuscript. After consulting the relevant literatures, we have revised our paper according to your suggestions and questions. Our answer as follows:

**Response to Editor:**

Our answer to question one *(Please respond to the reviewers' comments in a point-by-point manner*. In the abstract, "15 factors" is better to be written as "Fifteen factors...". In the abstract, "15 factors" is better to be written as "Fifteen factors..."*Thank you for revising the article according the suggestions of statistical reviewer. And, the statistical reviewer would like the authors to check the relationship between the results of Tables 4 and 5, and the logistic regression models described in Results. I think Tables 4 and 5 showed the prediction of pCR based on variables listed in Tables, however, P=logistic regression model in Results showed the formula of prediction of not pCR. How do the authors think?Thank you for revising the article according the suggestions of statistical reviewer. And, the statistical reviewer would like the authors to check the relationship between the results of Tables 4 and 5, and the logistic regression models described in Results. I think Tables 4 and 5 showed the prediction of pCR based on variables listed in Tables, however, P=logistic regression model in Results showed the formula of prediction of not pCR. How do the authors think?)* is as follows:

Thank you very much for your advice. In this reply, we give a point-to-point response to each comment of the reviewers.

Our answer to question two *(The authors wrote that "the tumor stem cell marker CD44v6, which has been reported to be related to the resistance of neoadjuvant chemoradiation in rectal cancer [5]." The writing was not clear for readers who are not working in this field. Please add one paragraph (or several sentences) to briefly introduce the role of CD44v6 in colorectal cancer and describe the reason(s) why the study chose this factor.*Thank you very much for your revision.
However, as mentioned above, our statistical advisor still has a small question. Can you check it carefully?
I also ask you to take care of significant digits again, especially in results section and tables.
Example: Materials and methods, Volumetric imaging parameters, line 4
rs=0.989
Why you show us three digits? Is there any meaning in the last digit “9”?*Thank you for revising the article according the suggestions of statistical reviewer. And, the statistical reviewer would like the authors to check the relationship between the results of Tables 4 and 5, and the logistic regression models described in Results. I think Tables 4 and 5 showed the prediction of pCR based on variables listed in Tables, however, P=logistic regression model in Results showed the formula of prediction of not pCR. How do the authors think?Thank you for revising the article according the suggestions of statistical reviewer. And, the statistical reviewer would like the authors to check the relationship between the results of Tables 4 and 5, and the logistic regression models described in Results. I think Tables 4 and 5 showed the prediction of pCR based on variables listed in Tables, however, P=logistic regression model in Results showed the formula of prediction of not pCR. How do the authors think?)* is as follows:

Thank you very much for your reminding. In the introduction of this revised manuscript, we added a paragraph to describe the relationship between the expression of CD44v6 and the patient's response to radiation.

**Response to Reviewer#1:**

Our answer to question one *(line 36-->"preoperative CCRT will result"-->I would change into" preoperative CCRT may result". In the abstract, "15 factors" is better to be written as "Fifteen factors...". In the abstract, "15 factors" is better to be written as "Fifteen factors..."Thank you for revising the article according the suggestions of statistical reviewer. And, the statistical reviewer would like the authors to check the relationship between the results of Tables 4 and 5, and the logistic regression models described in Results. I think Tables 4 and 5 showed the prediction of pCR based on variables listed in Tables, however, P=logistic regression model in Results showed the formula of prediction of not pCR. How do the authors think?Thank you for revising the article according the suggestions of statistical reviewer. And, the statistical reviewer would like the authors to check the relationship between the results of Tables 4 and 5, and the logistic regression models described in Results. I think Tables 4 and 5 showed the prediction of pCR based on variables listed in Tables, however, P=logistic regression model in Results showed the formula of prediction of not pCR. How do the authors think?)* is as follows:

Thank you very much for your reminding. We corrected this error in the revised manuscript.

Our answer to question two *(*line 51--> should be August 2017*. In the abstract, "15 factors" is better to be written as "Fifteen factors...". In the abstract, "15 factors" is better to be written as "Fifteen factors..."Thank you for revising the article according the suggestions of statistical reviewer. And, the statistical reviewer would like the authors to check the relationship between the results of Tables 4 and 5, and the logistic regression models described in Results. I think Tables 4 and 5 showed the prediction of pCR based on variables listed in Tables, however, P=logistic regression model in Results showed the formula of prediction of not pCR. How do the authors think?Thank you for revising the article according the suggestions of statistical reviewer. And, the statistical reviewer would like the authors to check the relationship between the results of Tables 4 and 5, and the logistic regression models described in Results. I think Tables 4 and 5 showed the prediction of pCR based on variables listed in Tables, however, P=logistic regression model in Results showed the formula of prediction of not pCR. How do the authors think?)* is as follows:

Thank you for your advice. We corrected this error in the revised manuscript.

Our answer to question three *(line 117-120. I have doubts about terminology of surgical procedures and surgical technique: Miles technique=abdominoperineal resection is always associated with end-colostomy formation,Dixon surgery-a type of blunt dissection"Until the late 1970s, anterior resection with blunt dissection of the mid and distal rectum (as described by Dixon) continued to have a disease-free five-year survival rate for all stages treated with curative intent not exceeding 50% with a local recurrence rate of up to 20%. This was mainly related to the breaches often created on the mesorectal fascia and the mesorectum itself during the blunt rectal dissection". Nowadays a standard procedure is TME introduced be RJ Heald-"A surgical plane is a “potential space between contiguous organs which can be reproducibly created by dissection” . In rectal surgery the plane develops between the mesorectum and the surrounding somatic structures . Dissection along this plane should be sharp, under direct vision and gentle continuous traction".*

*Question about ileostomy-->usually the temporary protective loop ileostomy is performed, not a terminal ileostomy.*

*Article about the terminology and technique-Minim Invasive Ther Allied Technol 25 (5), 226-33 Oct 2016 Techniques and Technology Evolution of Rectal Cancer Surgery: A History of More Than a Hundred Years Marco Maria Lirici , Cristiano G S Hüscher. In the abstract, "15 factors" is better to be written as "Fifteen factors...". In the abstract, "15 factors" is better to be written as "Fifteen factors..."Thank you for revising the article according the suggestions of statistical reviewer. And, the statistical reviewer would like the authors to check the relationship between the results of Tables 4 and 5, and the logistic regression models described in Results. I think Tables 4 and 5 showed the prediction of pCR based on variables listed in Tables, however, P=logistic regression model in Results showed the formula of prediction of not pCR. How do the authors think?Thank you for revising the article according the suggestions of statistical reviewer. And, the statistical reviewer would like the authors to check the relationship between the results of Tables 4 and 5, and the logistic regression models described in Results. I think Tables 4 and 5 showed the prediction of pCR based on variables listed in Tables, however, P=logistic regression model in Results showed the formula of prediction of not pCR. How do the authors think?)* is as follows:

Thank you very much for your reminding. After carefully reading the article you recommended, we modified the non-standard description in our article. At present, TME operation has been widely used in mainland China, but most clinicians still use the original operation name in their daily work, for example, the APR operation is called Miles, and the low anterior resection is called Dixon. This habitual title is not rigorous. Your reminder is very meaningful to us. We will correct it in our future clinical work and scientific research writing. In addition, in our revised version, terminal ileostomy was changed to temporary protective loop ileostomy. Thanks again!

**Response to Reviewer#2:**

Our answer to question one *(Table 1 has not been moved to the Results Scetion. In the abstract, "15 factors" is better to be written as "Fifteen factors...". In the abstract, "15 factors" is better to be written as "Fifteen factors..."Thank you for revising the article according the suggestions of statistical reviewer. And, the statistical reviewer would like the authors to check the relationship between the results of Tables 4 and 5, and the logistic regression models described in Results. I think Tables 4 and 5 showed the prediction of pCR based on variables listed in Tables, however, P=logistic regression model in Results showed the formula of prediction of not pCR. How do the authors think?Thank you for revising the article according the suggestions of statistical reviewer. And, the statistical reviewer would like the authors to check the relationship between the results of Tables 4 and 5, and the logistic regression models described in Results. I think Tables 4 and 5 showed the prediction of pCR based on variables listed in Tables, however, P=logistic regression model in Results showed the formula of prediction of not pCR. How do the authors think?)* is as follows:

According to your opinion, we moved Table 1 to the result section and briefly described it in the revised manuscript.

Our answer to question two *(The role of CD44v6 is still little described in Introduction. In the abstract, "15 factors" is better to be written as "Fifteen factors...". In the abstract, "15 factors" is better to be written as "Fifteen factors..."Thank you for revising the article according the suggestions of statistical reviewer. And, the statistical reviewer would like the authors to check the relationship between the results of Tables 4 and 5, and the logistic regression models described in Results. I think Tables 4 and 5 showed the prediction of pCR based on variables listed in Tables, however, P=logistic regression model in Results showed the formula of prediction of not pCR. How do the authors think?Thank you for revising the article according the suggestions of statistical reviewer. And, the statistical reviewer would like the authors to check the relationship between the results of Tables 4 and 5, and the logistic regression models described in Results. I think Tables 4 and 5 showed the prediction of pCR based on variables listed in Tables, however, P=logistic regression model in Results showed the formula of prediction of not pCR. How do the authors think?)* is as follows:

Thank you for your advice. In the introduction of this revised manuscript, we added a paragraph to describe the relationship between the expression of CD44v6 and the patient's response to radiation.

Our answer to question three *(Response to my fourth question remains unclear. Please provide a clear respsonse and define in Methods whether poor response was defined according to preoperative MRI or to pathological examination.. In the abstract, "15 factors" is better to be written as "Fifteen factors...". In the abstract, "15 factors" is better to be written as "Fifteen factors..."Thank you for revising the article according the suggestions of statistical reviewer. And, the statistical reviewer would like the authors to check the relationship between the results of Tables 4 and 5, and the logistic regression models described in Results. I think Tables 4 and 5 showed the prediction of pCR based on variables listed in Tables, however, P=logistic regression model in Results showed the formula of prediction of not pCR. How do the authors think?Thank you for revising the article according the suggestions of statistical reviewer. And, the statistical reviewer would like the authors to check the relationship between the results of Tables 4 and 5, and the logistic regression models described in Results. I think Tables 4 and 5 showed the prediction of pCR based on variables listed in Tables, however, P=logistic regression model in Results showed the formula of prediction of not pCR. How do the authors think?)* is as follows:

Your opinion is reasonable. We added this part in the materials and methods of this revised manuscript, and described the relevant results in our results section.

Our answer to question four *(Response to my fifth question is again approximate. I suggest to add to Limitations that an external cohort could be useful to validate this results and would strengthen your results.. In the abstract, "15 factors" is better to be written as "Fifteen factors...". In the abstract, "15 factors" is better to be written as "Fifteen factors..."Thank you for revising the article according the suggestions of statistical reviewer. And, the statistical reviewer would like the authors to check the relationship between the results of Tables 4 and 5, and the logistic regression models described in Results. I think Tables 4 and 5 showed the prediction of pCR based on variables listed in Tables, however, P=logistic regression model in Results showed the formula of prediction of not pCR. How do the authors think?Thank you for revising the article according the suggestions of statistical reviewer. And, the statistical reviewer would like the authors to check the relationship between the results of Tables 4 and 5, and the logistic regression models described in Results. I think Tables 4 and 5 showed the prediction of pCR based on variables listed in Tables, however, P=logistic regression model in Results showed the formula of prediction of not pCR. How do the authors think?)* is as follows:

Thank you for your reminding. We added this part to the Limitations of this revised manuscript. The details are as follows: The third limitation of this study is that the results we obtained have not appeared in the previous reports, plus there are fewer patients in this study (79 cases), so a larger cohort study still needs to be carried out. In addition, the practicability of our results also needs to be further verified in clinical practice.

Our answer to question five *(Figure 2 is unreadable.. In the abstract, "15 factors" is better to be written as "Fifteen factors...". In the abstract, "15 factors" is better to be written as "Fifteen factors..."Thank you for revising the article according the suggestions of statistical reviewer. And, the statistical reviewer would like the authors to check the relationship between the results of Tables 4 and 5, and the logistic regression models described in Results. I think Tables 4 and 5 showed the prediction of pCR based on variables listed in Tables, however, P=logistic regression model in Results showed the formula of prediction of not pCR. How do the authors think?Thank you for revising the article according the suggestions of statistical reviewer. And, the statistical reviewer would like the authors to check the relationship between the results of Tables 4 and 5, and the logistic regression models described in Results. I think Tables 4 and 5 showed the prediction of pCR based on variables listed in Tables, however, P=logistic regression model in Results showed the formula of prediction of not pCR. How do the authors think?)* is as follows:

Thank you for your reminding. In this picture upload, we divided figure 2 into Fig.2 and Fig.3.

Thank you and best regards.

Yours sincerely,

The author of PONE-D-19-31144

April 21, 2020

From the Editor:
Although the Vice Editor-In-Chief requested the corrections of "a significant figure" in the previous decision letter, authors did not revise them in the revised manuscript.
Abstract Line 5 from bottom: …the negative predictive values of TC were 74.7347%, 94.6097%, respectively.Please keep attention to the number of significant figures.
There are only 79 patients in your study. Therefore, they should be “75 and 95 %, respectively.
In total, 62 (78.48%) patients underwent low abdominal resection, and 17 (21.52%) underwent…78.46% should be 78% 21.52% should be 22% There are many this kind of errors in the following text.
Please correct all of them carefully again.From the Editor:
Although the Vice Editor-In-Chief requested the corrections of "a significant figure" in the previous decision letter, authors did not revise them in the revised manuscript.
Abstract Line 5 from bottom: …the negative predictive values of TC were 74.7347%, 94.6097%, respectively.Please keep attention to the number of significant figures.
There are only 79 patients in your study. Therefore, they should be “75 and 95 %, respectively.
In total, 62 (78.48%) patients underwent low abdominal resection, and 17 (21.52%) underwent…78.46% should be 78% 21.52% should be 22% There are many this kind of errors in the following text.
Please correct all of them carefully again.
